# Inhibition of lncRNA PCAT19 promotes breast cancer proliferation

**DOI:** 10.1002/cam4.5872

**Published:** 2023-03-29

**Authors:** Jianyuan Feng, Jiarui Zhang, Yanling Li, Weilun Cheng, Yansong Liu, Ziang Chen, Yunqiang Duan, Tianshui Yu, Anbang Hu, Ting Wang, Hanyu Zhang, Mingcui Li, Zhiyuan Rong, Fei Ma, Baoliang Guo

**Affiliations:** ^1^ Department of General Surgery The Second Affiliated Hospital of Harbin Medical University Harbin 150081 China

**Keywords:** bioinformatics, breast cancer, long noncoding RNA, tumor biology

## Abstract

**Background:**

Breast cancer (BC) is the most common malignancy affecting women. It is vital to explore sensitive biological markers to diagnose and treat BC patients. Recent studies have proved that long noncoding RNAs (lncRNAs) were involved in breast tumor progression. Nonetheless, whether lncRNA prostate cancer‐associated transcript 19 (PCAT19) impacts BC development remains unknown.

**Methods:**

We performed various bioinformatic analyses, including machine learning models to identify critical regulatory lncRNAs affecting prognosis in BC. The in situ hybridization (ISH) assay was carried out to confirm the expression levels of lncRNA PCAT19 in tissue specimens. MTT assay, wound healing assay, and transwell assay were performed to investigate PCAT19's impact on proliferation, migration, and invasion of BC cells. Mouse xenografts were used to examine the proliferation‐inhibiting function of PCAT19 in vivo.

**Results:**

Among the prognosis‐associated lncRNAs, PCAT19 predicted a favorable prognosis in BC. Patients with high expression levels of PCAT19 had a lower clinical stage and less lymph node metastasis. The PCAT19‐related genes were enriched in signaling pathways involved in tumor development, indicating PCAT19 was an essential regulator of BC. Using the ISH assay, we confirmed the expression level of lncRNA PCAT19 in human BC tissues was lower than normal breast tissues. Moreover, the knockdown of PCAT19 further confirmed its inhibiting ability in BC cell proliferation. Correspondingly, overexpressing PCAT19 reduced tumor size in mouse xenografts.

**Conclusions:**

Our study demonstrated that lncRNA PCAT19 suppressed the development of BC. PCAT19 might be a promising prognostic biomarker, which provides new insights into risk stratification for BC patients.

## Graphical abstract

This work demonstrated that lncRNA PCAT19 could curb breast cancer development by inhibiting proliferation. LncRNA PCAT19 is a potential biomarker of breast cancer and might help to improve precise patient stratification in the future.

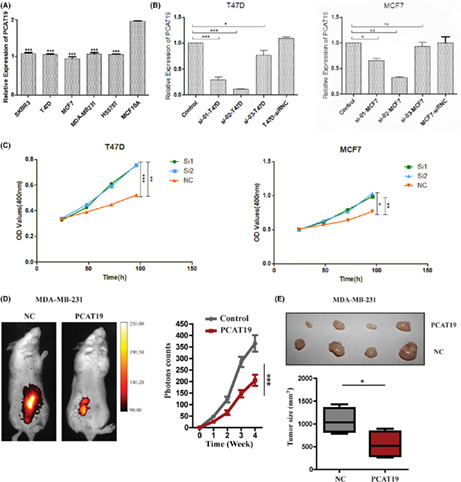

## 1 INTRODUCTION

Breast cancer (BC) is the leading cause of cancer‐related mortality, and it is also the most commonly diagnosed cancer among women worldwide.^1^ To decrease the disease burden of BC, there is an urgent need to explore the underlying mechanisms of tumorigenesis and identify potential therapeutic targets.

Long noncoding RNAs (lncRNAs), defined as RNAs longer than 200 nucleotides that lack open reading frames, are widely expressed in the human genome. Statistics from previous research suggested that there were more than 16,000 human lncRNA genes.^2^ LncRNAs are involved in a myriad of biological processes, both in psychological and disease conditions, as reviewed in detail by Statello et al.^3^ Briefly, the modulating mechanism of LncRNAs can be categorized into three aspects in relation to different levels of gene expression: chromatin regulation, transcriptional regulation, and post‐transcriptional regulation. In transcription/post‐transcription levels, lncRNAs can function both in cis or in trans, meaning targeting neighboring genes or distal genes. LncRNAs contribute to the hallmarks of cancer and serve as attractive potential biomarkers and therapeutic targets.^4^ In the cancer context, dysregulation of lncRNAs has been demonstrated to be closely related to cancer development. LncRNAs show regulating ability in both oncogenic and tumor‐suppressive pathways such as the p53, MYC, and NF‐kB pathways.^5^ LncRNAs play a regulatory role via a series of mechanisms, including acting as guides to direct chromatin‐modifying complexes to target genes (guide), as structural platforms for the assembly of multiple‐component complexes (scaffold), and binding to microRNAs or transcription factors to sequester them away from their targets (decoy), etc.^6^ Extensive studies have validated specific LncRNAs as hub modulators and prominent risk factors in multiple cancer types, such as MALAT1 and HOTAIR in lung cancer,^7^, ^8^ and LUNAR1 in acute leukemia.^9^

LncRNA PCAT19 (prostate cancer‐associated transcript 19), located at 19q13, was first found in prostate cancer (PCa).^10^ The expression of PCAT19 was elevated in PCa mediated by PCa‐risk‐associated SNP rs11672691, which mapped to the enhancer of PCAT19. Moreover, high expression of PCAT19 predicted relapse and worse survival in PCa.^11^ A previous meta‐analysis also reported that lncRNA PCAT19 was associated with PCa predisposition and PCa aggressiveness.^12^ Hua et al. found that PCAT19 activated cell cycle‐related genes by interacting with HNRNPAB, thus promoting PCa tumor growth and metastasis.^13^ Studies also found that PCAT19 contributed to carcinogenesis across other cancer types, including lung carcinoma, larynx carcinoma and glioma.^14^, ^15^, ^16^ However, there is currently no evidence concerning the effect of PCAT19 on BC. Our study found that PCAT19 was downregulated in BC patients and associated with a favorable prognosis. We further investigated the correlation between expression levels of LncRNA PCAT19 and the clinicopathological details of BC patients, as well as the mechanisms underlying the breast tumor‐inhibiting ability of PCAT19. In conclusion, our results suggested that lncRNA PCAT19 was crucially involved in BC progression and metastasis, which may provide a novel biomarker for BC.

## 2 MATERIALS AND METHODS

### 2.1 Dataset

The gene expression profiles and clinicopathological data of TCGA‐BRCA were accessed through NCI's Genomic Data Commons (GDC) (https://portal.gdc.cancer.gov/).

### 2.2 Machine learning model construction

Different machine learning models, including gradient boosting (GBM), random forest (RF), and support vector machine (SVM) classifiers, were implemented in Jupyter Notebook (version 6.3.0). Scikit‐learn module in Python (version 3.8.8) programming was adopted.

### 2.3 Differential analysis and functional enrichment analysis

The R package limma was used for identifying differentially expressed lncRNAs with statistical criteria setting at absolute fold change >2.0 and *p*‐value <0.01. Gene Ontology (GO), Kyoto Encyclopedia of Genes and Genomes (KEGG), and Reactome pathway enrichment analyses were carried out using the R package clusterProfiler (4.3.4), and the threshold of *p*‐value was set as <0.05.

### 2.4 ceRNA network construction

Predicted target mRNAs of PCAT19 were downloaded from LncExpDB (https://ngdc.cncb.ac.cn/lncexpdb/). Spearman's rank correlation coefficient was calculated between miRNA and PCAT19/target mRNAs, respectively. The miRNAs related to PCAT19 and target mRNAs were included in the ceRNA network and visualized in Cytoscape (3.9.1).

### 2.5 Patients and tissue specimens

A total of 151 BC tissues and 45 normal breast samples were obtained from the Department of Breast Surgery, The Second Affiliated Hospital of Harbin Medical University (246 Xuefu Street, Nangang District, Harbin, China). Written informed consent was acquired from all patients who participated in this study, and the research was approved by the Ethics Committee of Harbin Medical University. Fresh breast tissues were frozen in liquid nitrogen immediately after surgical excision and stored in a freezer at −80°C. Hematoxylin and eosin (H&E) staining was used for histologic validation of tissue types. Matched clinical and pathological data were properly recorded and stored in our department.

### 2.6 Cell culture

Human BC cell lines, including SKBR3, T47D, MCF7, MDA‐MB231, HS578T, and normal breast epithelial cell line MCF10A were incubated under the conditions that recommended by American Type Culture Collection. The culture media (Hyclone) were supplemented with 10% fetal bovine serum (FBS). All cells were cultured in a humidified atmosphere containing 5% CO_2_ at 37°C.

### 2.7 In situ hybridization (ISH) assay

The expression level of lncRNA PCAT19 of all paraffin‐embedded tissue samples was evaluated with the ISH Kit (BOSTER, catalog number: MK1030). The ISH assays were performed according to the manufacturer's procedure. Briefly, the paraffin‐embedded tissue sections were dewaxed, rehydrated and then digested with protease K followed by rinsing with PBS. Afterwards, the buffer containing the probes was added and incubated at 65°C for 12–16 h. The coverslip was washed with PBS and fixed in 4% paraformaldehyde.

### 2.8 RNA extraction and RT‐PCR

Total RNA was extracted and reverse transcribed by TRIZOL reagent (Invitrogen). 18sRNA was selected as the reference gene because of the highest expression stability calculated by the NormFinder algorithm. LncRNA PCAT19 was detected using Platinum Taq DNA Polymerase (Invitrogen) with specific primers: LncRNA PCAT19 forward: AGTTTCCTGACCTACTCTGCTGC, LncRNA PCAT19 reverse: TCAATGCTTTTCTTCCCCGA, and 18sRNA forward: GTCGCTCGCTCCTCTCCTACTT, 18sRNA reverse: CTGATAAATGCACGCATCCCC. Results were expressed as fold change using the 2‐CT method.

### 2.9 Plasmid construction and transfection

For overexpression experiments, lentiviral plasmid (pLVX‐puro) containing full‐length sequence of human PCAT19 and control plasmid were generated. For the transfection of siRNA PCAT19, siRNA PCAT19 sequence were amplified from LncRNA PCAT19 construct (System Biosciences) and cloned into pSilencer4.1 system. BC cells were then transfected with the pSliencer vector containing the antisense sequence of LncRNA PCAT19. The cells were cultured with puromycin after 48 h of transfection to filter the stable cell lines. Control, si‐01, si‐02, si‐03, and non‐targeted control siRNA (siRNC) of BC cells were purchased from Genepharma. The cDNA ORF clones were purchased from Origene Technology. Transient transfections of plasmids and siRNAs were performed using Lipofectamine 3000 (Invitrogen) following the manufacturer's protocol. Cells were kept in a medium containing 2% FBS for 48 h and then harvested and used.

### 2.10 Cell proliferation assay

After the transfection of si‐LncRNA PCAT19, the proliferation of T47D and MCF7 cancer cells was determined by the MTT assay according to provided protocols. The absorbance at 400 nm was measured for each sample. The cell proliferation rate was calculated based on the OD400 value.

### 2.11 Wound healing assay

To measure cell migration, T47D and MCF7 cells were seeded in 35‐mm dishes and cultured for 1 day. A scraped line was created on the surface of the dishes with a 200‐μl pipette tip when the cells reached approximately 100% confluence. Then the medium was replaced and the cells were cultured for 24 h. The wound area was photographed with an inverted microscope (Carl Zeiss), and the wound closure was quantified utilizing the Image J software.

### 2.12 Matrigel invasion assay

To investigate the cell invasion ability, the transwell assay was performed with 24‐well Matrigel‐coated invasion chambers (Corning). Briefly, the stable monoclonal cell lines (2.0 × 10^5^ cells per well) were resuspended in the medium without FBS and placed in the upper compartment of transwell chambers with fibronectin coated on the lower surface. The lower compartment was filled with 700 μL medium containing 10% FBS as a chemoattractant. Cells were fixed in 4% formaldehyde and stained with 0.1% crystal violet after 24 h. Ten fields were randomly selected and counted under a light microscope (Carl Zeiss).

### 2.13 Animal studies

All animal studies were ratified by the ethics committee of Harbin Medical University (Harbin, China). The MDA‐MB231 cells were injected in the flank of 4‐week‐old female mice (each group has four mice). Tumor volume was tracked every week by intraperitoneal injection of D‐luciferin followed by Xenogen IVIS Spectrum Imaging System (Caliper Life Sciences), with tumor volume quantified by Living Image Software. The mice were euthanized after 4 weeks by ip injection of sodium pentobarbital (200 mg/kg), and the primary tumors was removed and measured.

### 2.14 Statistical analysis

The differences in MTT assay, wound healing assay, and transwell migration assay between cells transfected with si‐PCAT19 and scrambled oligonucleotides control were compared by Student's *t*‐test. The chi‐squared test was used to analyze the associations between LncRNA PCAT19 expression and various prognostic factors. The Kaplan–Meier method and log‐rank test were used to calculate survival analysis. The statistical analyses mentioned above were performed using SPSS software version 24.0 (SPSS, Inc). Univariate and multivariate Cox regression analysis was performed in R (4.2.0). Statistical significance was defined as a *p*‐value <0.05.

## 3 RESULTS

### 3.1 LncRNA PCAT19 was downregulated in BC and predicted a better prognosis

**FIGURE 1:**
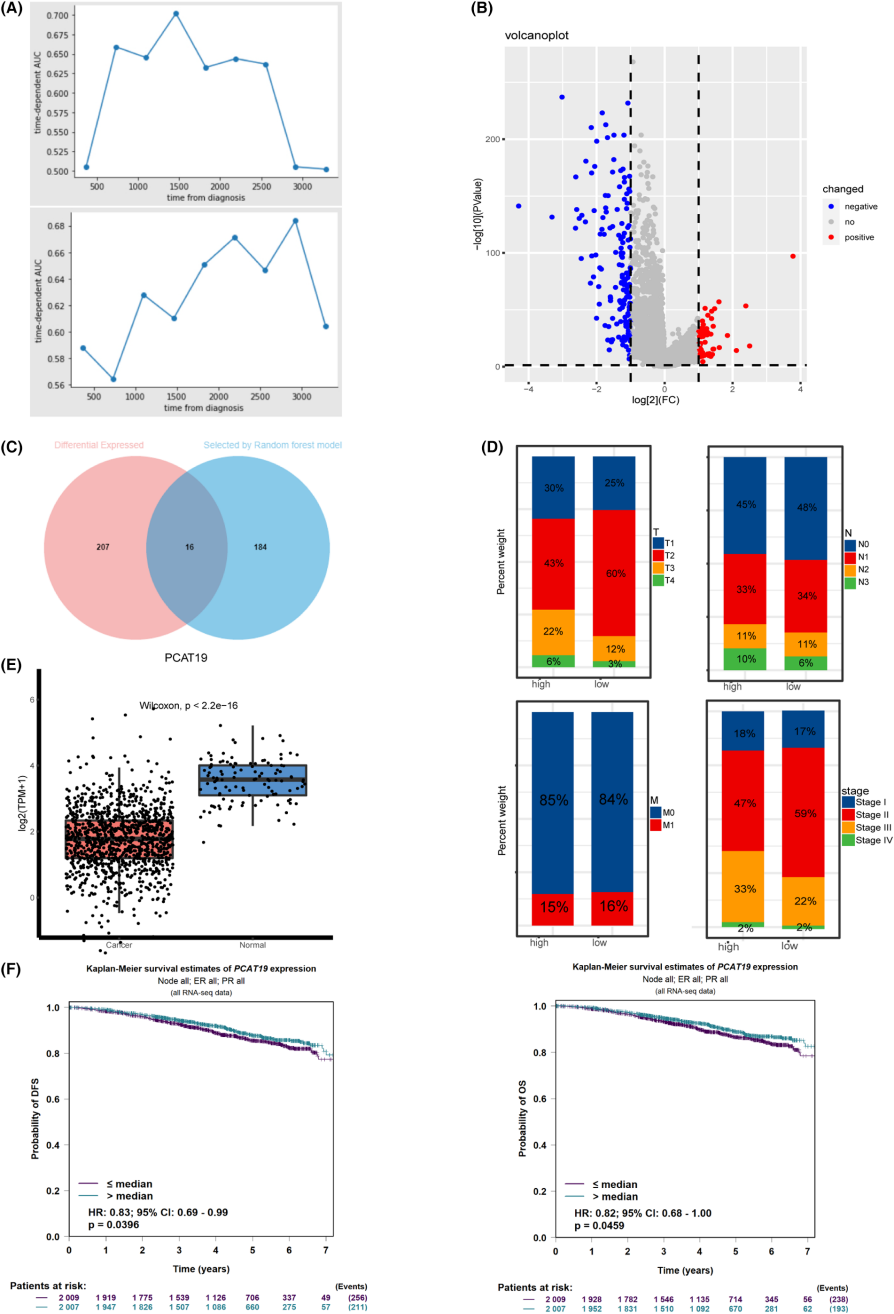
High expression of LncRNA PCAT19 correlated positively with longer overall survival. A. Time‐dependent AUC of RF model (upper) and gradient boosting model (bottom); B. Volcano plot of differential analysis using lncRNAs expression profiles in TCGA‐BRCA. The blue dots represent lncRNAs downregulated in tumor samples and the red dots stand for upregulated lncRNAs in BC; C. Venn plot shows the intersection of lncRNAs statistically significant in differential analysis (left) and selected by RF model (right); D. Distribution of T stage (upper left), N stage (upper right), M stage (bottom left) and clinical stage (bottom right) between the high‐expression group and low‐expression group; F. Kaplan–Meier analysis of PCAT19 (cutoff was set as a median expression) using data of DFS (left) and OS (right); Abbreviations, AUC, area under curve; BC, breast cancer; DFS, disease‐free survival; RF, random forest; OS, overall survival.

To investigate the potential impact of lncRNAs on BC prognosis, lncRNA expression profiles of 1076 BC patients and 99 normal controls were downloaded from TCGA with matched survival data. After removing samples with 0 days of observation time, 1054 BC samples were put into the machine learning analysis. Four models were used to examine the association between lncRNAs and overall survival (OS), including the RF survival model, GBM, linear survival SVM, and kernel survival SVM. The RF survival model showed the best predictability among all models (C‐index of linear survival SVM = 0.632, C‐index of kernel survival SVM = 0.561) (Figure 1A). Therefore, lncRNAs that were ranked in the top 10% of feature importance using RF survival model were selected for subsequent analysis. Next, we performed differential analysis between normal and cancer patients to identify possible carcinogenic lncRNAs. The results showed that 223 lncRNAs were differentially expressed in tumor patients with 58 upregulated and 165 downregulated lncRNAs (Figure 1B). We then focused on the 16 lncRNAs that were both differentially expressed and related to prognosis (LINC02544, C6orf99, MAFG‐DT, PCAT19, AL096828.3, LINC01235, AC009005.1, AC009686.2, CEBPA‐DT, HOXC‐AS1, AC022509.3, AP000547.3, AL512329.2, AC092306.1, MIR205HG, LINC02489) (Figure 1C). To validate the OS‐associated lncRNAs, we searched the bc‐GenExMiner database,^17^ which contains 4421 BC patients' RNA‐seq profiles and found that PCAT19 was a protective prognosis factor of BC as a whole cohort (*p*‐value for DFS = 0.039, *p*‐value for OS = 0.045) as well as in luminal A/B subtypes (Figure 1F, Figure S1). In concordance with this finding, PCAT19 was significantly downregulated in tumor samples and expressed higher in patients with lower tumor grade, clinical stage, and better lymph node status (Figure 1D,E).

### 3.2 LncRNA PCAT19 was associated with tumor‐suppressive genes

**FIGURE 2:**
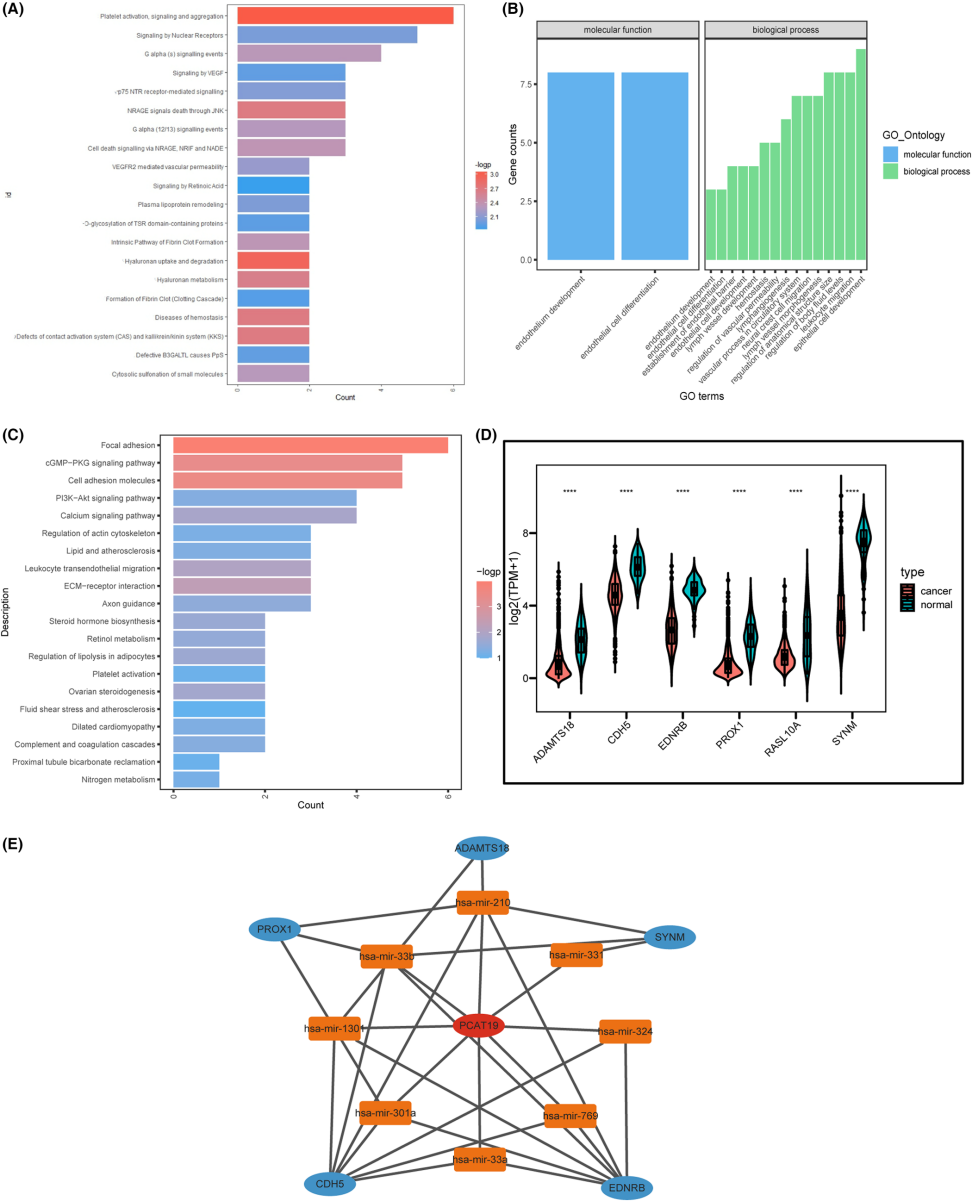
Enrichment analysis and ceRNA network of PCAT19‐associated genes A. Enrichment analysis of PCAT19‐targeted genes in Reactome database (*X* axis represents gene counts enriched in each pathway, *Y* axis represents pathway names); B. GO analysis of PCAT19‐targeted genes (*X* axis represents GO terms, *Y* axis represents gene counts enriched in each term); C. KEGG analysis of PCAT19‐targeted genes; D. Tumor‐suppressing genes that were downregulated in BC patients; E. ceRNA network of PCAT19‐associated miRNAs and PCAT19‐targeted tumor‐suppressing genes constructed by Cytoscape; Abbreviations, ceRNA, competing endogenous RNA; GO, gene ontology; KEGG, Kyoto encyclopedia of genes; *p < 0.05, **p < 0.01, ***p < 0.001, and ****p < 0.0001.

LncRNA PCAT19 has been demonstrated to be involved in tumorigenesis in multiple types of cancer.^13^, ^18^ To examine the function of PCAT19 in BC, we obtained the possible target mRNAs from LncExpDB and performed differential analysis followed by pathway enrichment analysis. Overall, there were 85 statistically differentially expressed target mRNAs, most of which were downregulated in tumor samples (Table S1). GO, KEGG, and Reactome enrichment analysis revealed that PCAT19‐target genes were enriched in biological processes essential for cancer development such as epithelial development, lymph vessel morphogenesis, regulation of vascular process and carcinogenesis pathways, including VEGF signaling pathway, PI3K‐AKT signaling pathway, and cGMP‐PKG signaling pathway^19^, ^20^ (Figure 2A–C). Previous studies have illustrated that competitive endogenous RNAs (ceRNA) played a significant role in lncRNA functional mechanisms.^21^ Due to the possible antitumor effect of PCAT19, we screened out six target genes (ADAMTS18, CDH5, EDNRB, PROX1, RASL10A, and SYNM) that were both downregulated in BC and had tumor‐suppressing ability with experimental support^22^ (Figure 2D). MicroRNAs (miRNA) negatively correlated with PCAT19 (miR‐210, miR‐331, miR‐324, miR‐769, miR‐33a, miR‐301a, miR‐1301, and miR‐33b) and six tumor‐suppressing genes were introduced to the construction of ceRNA network (Figure 2E).

### 3.3 LncRNA PCAT19 was lowly expressed in BC tissues and associated with pathological features

**FIGURE 3:**
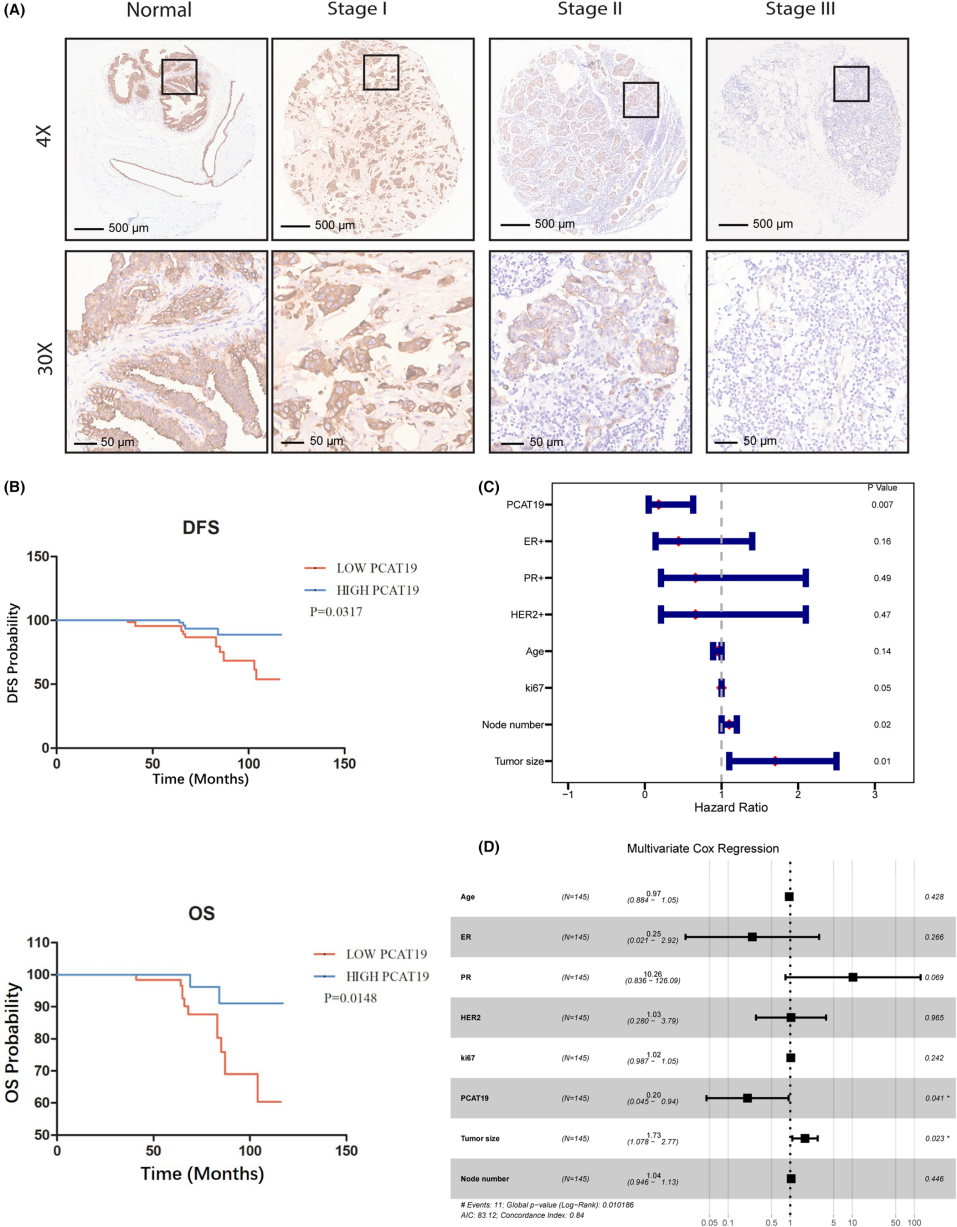
PCAT19 was downregulated in breast cancer (BC) tissues and was an independent prognosis factor A. The relative expression of long noncoding RNA prostate cancer‐associated transcript 19 (PCAT19) in breast cancer tissues across different clinical stages and normal breast tissues using in situ hybridization; B. The Kaplan–Meier curves for RFS and overall survival of BC patients stratified by PCAT19 expression level; C. Forest plot of univariate cox regression, the gray dashed line represents hazard ratio equals 1; D. Forest plot of multivariate cox regression, the black dashed line represents hazard ratio equals 1.

To further validate the expression of PCAT19 in BC patients, we performed ISH using a tissue microarray comprised of formalin‐fixed paraffin‐embedded tissues from patients who underwent surgery in our department. We found that lncRNA PCAT19 was localized in the cytoplasm and membrane of BC cells, and the expression of PCAT19 was higher in early‐stage BC patients (Figure 3A). Moreover, PCAT19 low‐expression patients had smaller tumor size and lower pathological grade, which indicate lower tumor burden and better prognosis (Table 1).

**TABLE 1:** Association between clinical features of the patients and LncRNA PCAT19 expression.

Parameters	Numbers of cases	LncRNA PCAT19		*p*‐Value
		low	high	
Age (years)				
<50	80	40	40	
≥50	71	35	36	0.931
Axillary lymph node				
Positive	92	38	54	
Negative	59	37	22	0.01
Pathological grade				
Grade 1	26	8	18	
Grade 2	64	25	39	
Grade 3	61	42	19	<0.01
Clinical stage				
Stage 1	89	28	61	
Stage 2	57	42	15	
Stage 3	5	5	0	<0.01
T stage				
T1	91	30	61	
T2	55	40	15	
T3	5	5	0	<0.01
PN				
N1	121	53	68	
N2	25	17	8	
N3	5	5	0	0.006
Molecular subtype				
TNBC	22	15	7	
Luminal	86	38	48	
HER‐2	43	22	21	0.129
ER				
Negative	53	32	21	
Positive	98	43	55	0.053
PR				
Negative	59	35	24	
Positive	92	40	52	0.057
HER‐2				
Negative	50	26	24	
Positive	101	49	52	0.678
Ki67 (%)				
<30	16	7	9	
≥30	135	68	67	0.617

The survival analysis proved that PCAT19‐low patients surpassed PCAT19‐high patients in both DFS and OS (Figure 3B). Furthermore, PACT19 expression was statistically significant in univariate and multivariate cox regression analyses, suggesting that PCAT19 was an independent prognosis factor in BC (Figure 3C,D).

### 3.4 LncRNA PCAT19 suppressed BC proliferation in vitro and in vivo

**FIGURE 4:**
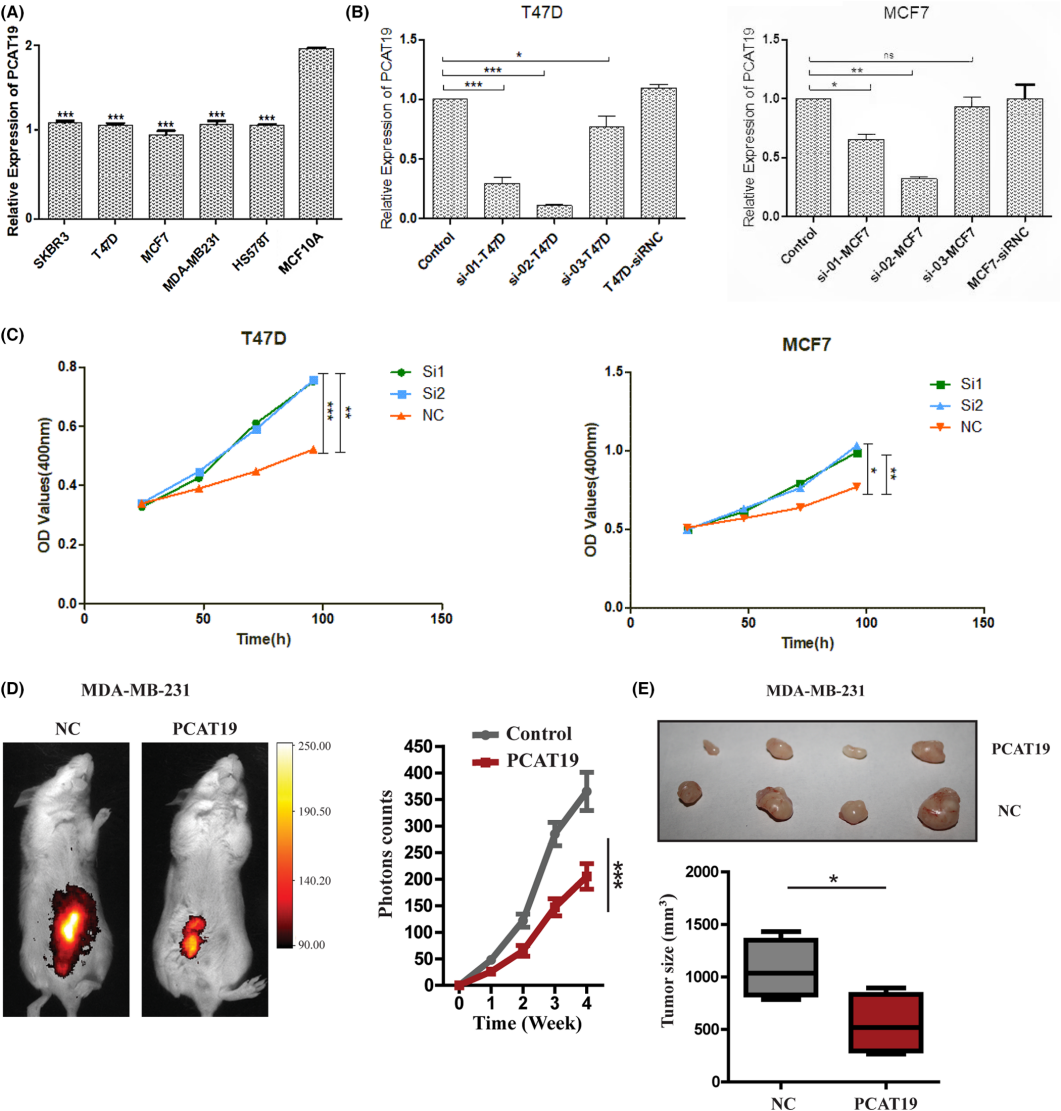
PCAT19 affected breast cancer proliferation in vitro and in vivo A. The expression of PCAT19 in all breast cancer cell lines; B. The expression level of lncRNA PCAT19 was decreased after siRNA interference; C. Knockdown of lncRNA PCAT19 promoted the growth of both MCF‐7 and T47D cells; D. Tumor growth was measured non‐invasively by IVIS imaging after administering luciferin for 4 weeks. The bioluminescence intensity of tumors was quantitated and presented as the mean of photon counts per second per tumor; E. Tumors were removed after 4 weeks. The tumor value was calculated with the equation *v* = (length*width^2^)/2; *p < 0.05, **p < 0.01, ***p < 0.001, and ****p < 0.0001.

Given the correlation of lncRNA PCAT19 expression and prognosis in BC patients, we next explored the effect of lncRNA PCAT19 on BC cells. Compared with normal breast epithelial cells, PCAT19 expression level was lower in cancer cells (Figure 4A). We stably transfected T47D and MCF‐7 cell lines with control vector and knockdown vectors, and the expression of PCAT19 was confirmed by real‐time PCR. Three siRNAs designed to disrupt lncRNA PCAT19 expression specifically silenced PCAT19 (Figure 4B). The cell proliferation assay indicated that the knockdown of lncRNA PCAT19 promoted the growth of MCF‐7 and T47D cells (Figure 4C). The results of the wound healing assay and the Matrigel‐coated transwell assay showed no statistically significant change in migration/invasion between control and si‐PCAT19 cells. Despite the fact that the difference was not significant, depletion of PCAT19 still had the trend to enhance the migration and invasion of MCF‐7 and T47D cells in vitro (Figure S2). Consistent with the results of the proliferation assay in vitro, overexpression of PCAT19 reduced tumor growth in vivo (Figure 4D,E). Above all, we found that PCAT19 exerted tumor‐suppressing ability by inhibiting cancer cell proliferation in vitro and in vivo.

## 4 DISCUSSION

Recently, emerging evidence has revealed that alterations in lncRNAs were associated with tumor cell behavior and affected cancer invasion and metastasis in BC. By harnessing different functional mechanisms, lncRNAs regulate metastasis, epithelial‐to‐mesenchymal transition (EMT), and stemness in BC.^23^ Acting as miRNA sponges, PNUTS and LINC‐ZNF469‐3 promote lung metastasis through PNUTS/miR‐205 and LINC‐ZNF469‐3/miR‐574‐5p axis, respectively.^24^, ^25^ Functioning as a scaffold lncRNA, MAYA modulates BC bone metastasis via YAP‐dependent transcription.^26^ With regard to EMT, NEAT1 was proved to be a positive regulator of EMT, while MALAT1 executes EMT‐suppressing ability by forming a ribonucleoprotein complex, which binds to the promotor region of CD133.^27^, ^28^ Furthermore, several studies have unveiled that lncRNAs also affect drug resistance of BC. For instance, BORG and H19 contribute to chemotherapy resistance, while HOTAIR and TMPO‐AS1 are associated with tamoxifen resistance.^23^ Notably, there have been attempts to utilize lncRNAs as therapeutic targets for BC patients. Antisense oligonucleotides of MALAT1 and HOTAIR have shown effective tumor‐inhibiting power in preclinical models.^29^

In this study, we found that the expression of lncRNA PCAT19 was downregulated in BC and was a favorable prognostic factor using a patient cohort of 153 BC tissue samples with corresponding clinical pathological details and follow‐up data during the past 10 years. Our results proved that lncRNA PCAT19 inhibited BC cells' proliferation, suggesting lncRNA PCAT19 may serve as a potential prognostic indicator and therapeutic target for BC patients. Patients with low expression level of PCAT19 can be identified as high risk who need more radical treatments because of the poor prognosis. Up to date, no known therapeutic compound that directly targets PCAT19 has been found. Searching for such a compound and clarifying whether increase the expression of PCAT19 (e.g., inhibiting PCAT19‐associated miRNAs) affect BC growth in clinical setting is a promising field for future studies.

Admittedly, there are still limitations in our study. Our patient cohort was restricted by region and sample size. We are currently working on modifying the patient cohort by expanding the enrolled population and making the cohort more representative. Besides, using bioinformatic methods, we proposed several ceRNA regulatory axes possible for PCAT19 to exert cancer‐suppressing ability. The actual mechanisms of lncRNA PCAT19 in BC still wait to be further elucidated by in vitro and in vivo experiments.

Plentiful studies have emphasized the interaction between lncRNAs and miRNAs in cancer.^30^ Xu et al. illustrated that LncRNA PCAT19/ miR‐182/PDK4 axis regulated cell proliferation by modulating glycolysis and mitochondrial respiration in laryngeal cancer.^16^ In lung cancer, overexpression of LncRNA PCAT19 increased apoptosis in cancer cells through miR‐25‐3p/MAP2K4 signaling axis.^31^ In this study, we plotted a ceRNA network by implementing bioinformatic methods. Among the target genes in the network, silencing ADAMTS18 can aggravate the malignancy of BC via NF‐κB signaling. Downregulation of PROX1 and SYNM was also observed in BC tissues and correlated with worse survival.^32^, ^33^, ^34^ Accordingly, various miRNAs in the network exert oncogenic ability in BC. For example, miR‐210 can be induced by hypoxia and associates inversely to DFS of BC patients,^35^ and miR‐301a can promote BC metastasis by activating Wnt/β‐catenin signaling.^36^ More exploration of the mechanism underlying PCAT19's breast‐cancer‐suppressing function might uncover new axis of lncRNA‐miRNA interaction.

## 5 CONCLUSION

Our results demonstrated that lncRNA PCAT19 could curb BC development by inhibiting proliferation. LncRNA PCAT19 is a potential biomarker of BC and might help to improve precise patient stratification in the future.

## AUTHOR CONTRIBUTIONS

**Jianyuan Feng:** Formal analysis (lead); software (equal). **Jiarui Zhang:** Investigation (lead); resources (equal). **Yanling Li:** Formal analysis (equal); software (equal); writing – original draft (equal). **Weilun Cheng:** Validation (equal). **Yansong Liu:** Software (lead). **Ziang Chen:** Validation (equal). **Yunqiang Duan:** Validation (equal). **Tianshui Yu:** Writing – review and editing (equal). **Anbang Hu:** Data curation (lead); visualization (equal). **Ting Wang:** Writing – review and editing (equal). **Hanyu Zhang:** Resources (equal). **Mingcui Li:** Writing – original draft (equal). **Zhiyuan Rong:** Visualization (equal). **Fei Ma:** Conceptualization (equal); funding acquisition (equal); methodology (lead); project administration (equal). **Baoliang Guo:** Conceptualization (equal); funding acquisition (lead); project administration (lead).

## FUNDING INFORMATION

This research was supported by grants from the National Natural Science Foundation of China (81872135, 82002791) and the Funds for Distinguished Young Scientists of the Second Affiliated Hospital of Harbin Medical University.

## CONFLICT OF INTEREST STATEMENT

The authors declare that there is no conflict of interest regarding the publication of this article.

## ETHICS STATEMENT

This study was conducted with the approval of the Institutional Review Board at the Harbin Medical University (KY2018‐068).

## PATIENT CONSENT STATEMENT

Informed consent was obtained from the patients in this study.

## Supporting information


**Supporting information S1.** Supplementary material

## Data Availability

Publicly available datasets were analyzed in this study. This data can be found here: https://portal.gdc.cancer.gov/.
