# Supplementary material for: Inhibition of lncRNA PCAT19 promotes breast cancer proliferation
Source: Cancer Med. 2023 Mar 29;12(10):11971–82. doi: 10.1002/cam4.5872 (PMC10242319; doi:10.1002/cam4.5872)
Supplement: Supplementary file 1 — Supporting information S1. Supplementary material [file CAM4-12-11971-s001.docx]

| gene | log2FoldChange | pvalue | padj |
| --- | --- | --- | --- |
| CA4 | -5.39357 | 2.11E-82 | 4.93E-81 |
| LYVE1 | -5.07533 | 4.05E-257 | 5.10E-254 |
| FABP4 | -4.87999 | 2.24E-102 | 8.49E-101 |
| CYP1A1 | -4.02146 | 2.23E-47 | 1.97E-46 |
| PKHD1L1 | -3.63368 | 4.25E-79 | 9.08E-78 |
| PDK4 | -3.63019 | 3.95E-122 | 2.24E-120 |
| STAB2 | -3.55877 | 1.11E-75 | 2.13E-74 |
| GPIHBP1 | -3.51156 | 1.29E-117 | 6.70E-116 |
| MMRN1 | -3.43044 | 6.31E-89 | 1.71E-87 |
| GCOM1 | -3.25246 | 9.75E-139 | 8.09E-137 |
| PDE2A | -3.1933 | 2.04E-173 | 3.41E-171 |
| SYNM | -3.14805 | 1.21E-78 | 2.56E-77 |
| RELN | -2.97409 | 5.96E-42 | 4.42E-41 |
| SEMA3G | -2.85483 | 4.41E-128 | 2.93E-126 |
| GIPC2 | -2.73063 | 1.08E-101 | 4.02E-100 |
| AVPR2 | -2.70037 | 2.57E-77 | 5.19E-76 |
| CETP | -2.60134 | 8.14E-78 | 1.68E-76 |
| ALDH1A1 | -2.58361 | 1.30E-97 | 4.38E-96 |
| CLDN5 | -2.53149 | 4.62E-71 | 7.95E-70 |
| CCL21 | -2.4196 | 1.08E-31 | 5.68E-31 |
| CLEC4M | -2.34565 | 2.35E-28 | 1.09E-27 |
| PROX1 | -2.33349 | 4.61E-41 | 3.32E-40 |
| ITM2A | -2.24528 | 3.63E-69 | 5.94E-68 |
| EDNRB | -2.23183 | 1.20E-92 | 3.60E-91 |
| KLHL4 | -2.19373 | 7.96E-80 | 1.74E-78 |
| PLSCR4 | -2.17079 | 1.63E-125 | 1.02E-123 |
| SHE | -2.16081 | 1.39E-112 | 6.55E-111 |
| LRRC70 | -2.13029 | 1.39E-113 | 6.77E-112 |
| CD34 | -2.1259 | 1.94E-158 | 2.27E-156 |
| RASL10A | -2.12275 | 5.48E-59 | 6.92E-58 |
| VWF | -2.09635 | 2.14E-111 | 9.85E-110 |
| TDRD10 | -2.06236 | 1.18E-55 | 1.35E-54 |
| C2CD4B | -2.06158 | 2.25E-29 | 1.09E-28 |
| USHBP1 | -1.98769 | 1.57E-71 | 2.74E-70 |
| TAL1 | -1.97821 | 4.60E-95 | 1.45E-93 |
| SH3BGRL2 | -1.97118 | 8.00E-90 | 2.22E-88 |
| EMCN | -1.96261 | 5.24E-88 | 1.40E-86 |
| GIMAP8 | -1.90059 | 1.15E-101 | 4.26E-100 |
| MRC1 | -1.86973 | 1.16E-29 | 5.69E-29 |
| ARHGEF15 | -1.84085 | 5.23E-95 | 1.64E-93 |
| PPP1R16B | -1.83639 | 1.34E-46 | 1.16E-45 |
| F8 | -1.80391 | 2.11E-112 | 9.89E-111 |
| ART4 | -1.79179 | 3.92E-17 | 1.16E-16 |
| ECSCR | -1.78247 | 9.34E-81 | 2.09E-79 |
| KANK3 | -1.74001 | 2.52E-65 | 3.79E-64 |
| CLEC1A | -1.69015 | 5.97E-80 | 1.31E-78 |
| ADAMTS18 | -1.67928 | 4.07E-18 | 1.25E-17 |
| CDH5 | -1.64695 | 8.63E-88 | 2.29E-86 |
| SLC5A4 | -1.61151 | 5.61E-48 | 5.07E-47 |
| ARL4A | -1.57721 | 9.08E-64 | 1.30E-62 |
| RHOU | -1.5142 | 1.27E-40 | 9.08E-40 |
| SELE | -1.50913 | 3.23E-19 | 1.04E-18 |
| LONRF3 | -1.46364 | 8.56E-27 | 3.74E-26 |
| KCNIP1 | -1.44788 | 7.54E-19 | 2.38E-18 |
| GIMAP7 | -1.44561 | 1.54E-40 | 1.09E-39 |
| TRPC6 | -1.41644 | 1.78E-49 | 1.68E-48 |
| RASIP1 | -1.40778 | 8.01E-46 | 6.73E-45 |
| HSPA12B | -1.38886 | 3.35E-44 | 2.67E-43 |
| CRTAC1 | -1.38325 | 5.84E-11 | 1.30E-10 |
| SHANK3 | -1.35326 | 4.21E-65 | 6.31E-64 |
| ADRB1 | -1.33543 | 2.20E-09 | 4.49E-09 |
| SEMA6A | -1.32057 | 3.90E-25 | 1.60E-24 |
| ITGA9 | -1.30332 | 7.57E-29 | 3.57E-28 |
| GIMAP5 | -1.29923 | 6.58E-34 | 3.76E-33 |
| SULT1C4 | -1.25255 | 1.97E-16 | 5.63E-16 |
| C1orf115 | -1.20808 | 3.26E-26 | 1.40E-25 |
| FLT4 | -1.18829 | 6.82E-48 | 6.15E-47 |
| KALRN | -1.18002 | 5.50E-32 | 2.92E-31 |
| TIE1 | -1.17 | 2.71E-45 | 2.24E-44 |
| TSPAN11 | -1.09486 | 3.09E-18 | 9.55E-18 |
| HSD17B2 | -1.04372 | 2.41E-05 | 3.87E-05 |
| ADAMTSL3 | -1.03309 | 2.09E-15 | 5.73E-15 |
| SLC30A3 | 1.081243 | 2.68E-09 | 5.47E-09 |
| REEP1 | 1.183414 | 6.69E-16 | 1.87E-15 |
| TBX1 | 1.336121 | 4.64E-15 | 1.25E-14 |
| DYSFIP1 | 1.34769 | 1.50E-08 | 2.92E-08 |
| VAV3 | 1.351242 | 5.18E-28 | 2.37E-27 |
| CEACAM16 | 1.478153 | 4.59E-05 | 7.22E-05 |
| MYCN | 1.671006 | 1.01E-20 | 3.46E-20 |
| PODXL2 | 1.718433 | 1.65E-49 | 1.56E-48 |
| LAMP3 | 1.891239 | 4.21E-28 | 1.93E-27 |
| MYLK2 | 2.358019 | 1.11E-45 | 9.26E-45 |
| GNAT3 | 2.482517 | 1.91E-03 | 2.68E-03 |
| TFF3 | 2.587735 | 1.14E-29 | 5.60E-29 |
| KCNF1 | 3.423058 | 2.50E-55 | 2.84E-54 |

Table S1 PCAT19-targeted genes that were differentially expressed in BC

Figure S1


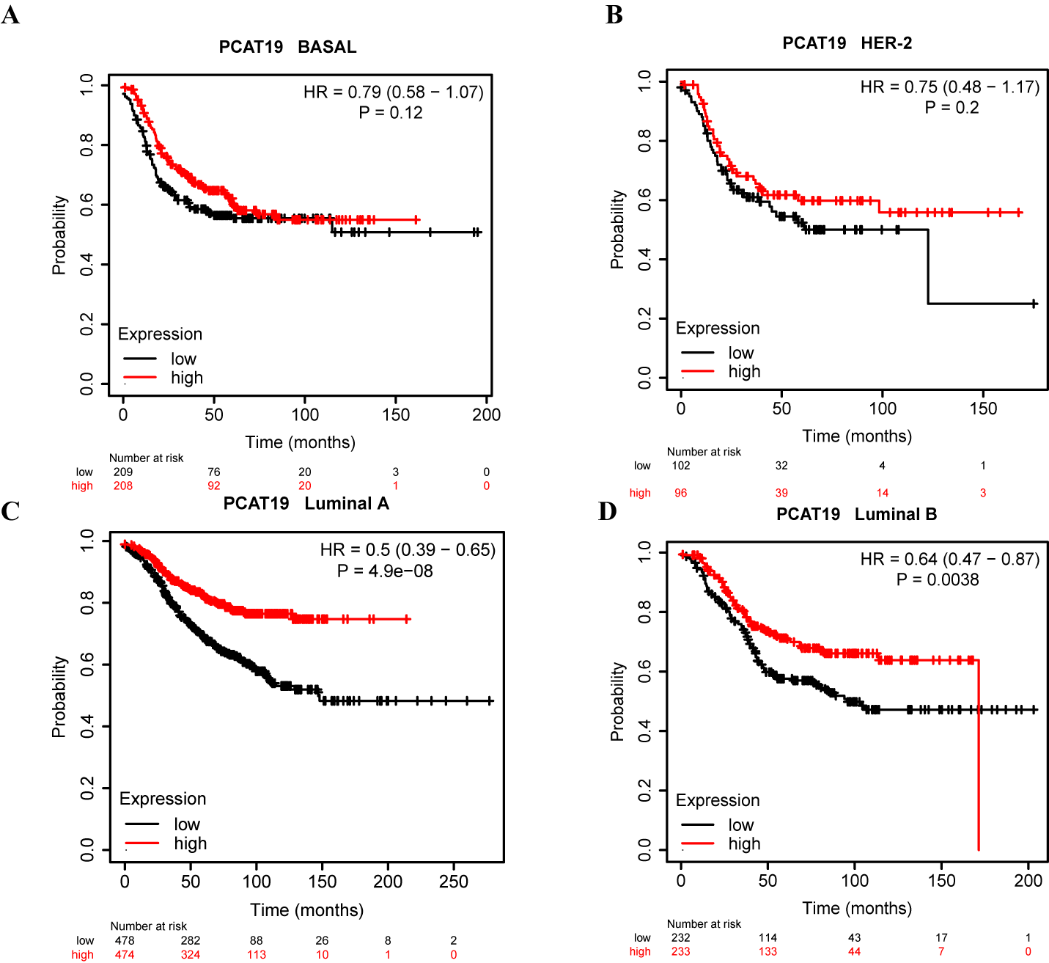


Figure S1 The impact of PCAT19 on overall survival in molecular subtypes

A. Kaplan-Meier survival analysis of PCAT19 expression in basal subtype; B. Kaplan-Meier survival analysis of PCAT19 expression in Her-2 subtype; C. Kaplan-Meier survival analysis of PCAT19 expression in luminal A subtype; D. Kaplan-Meier survival analysis of PCAT19 expression in luminal B subtype.

Figure S2


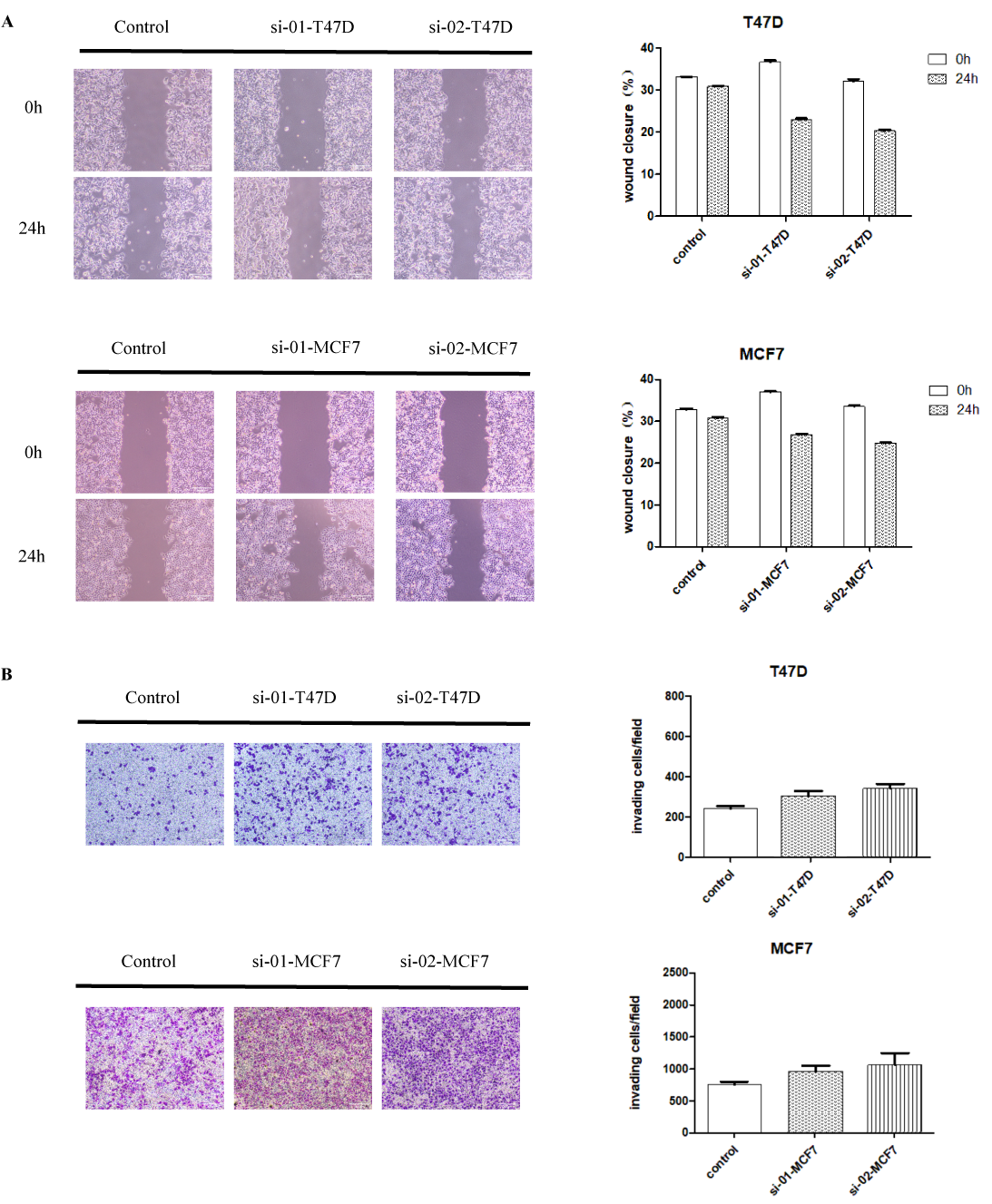


Figure S2 Knockdown of PACT19 did not significantly affect migration and invasion A. The percentage of wound closure changed insignificantly between si-PCAT19 and control; B. No significant alteration of invading ability was observed in transwell assay between control and si-PACT19

Figure S3


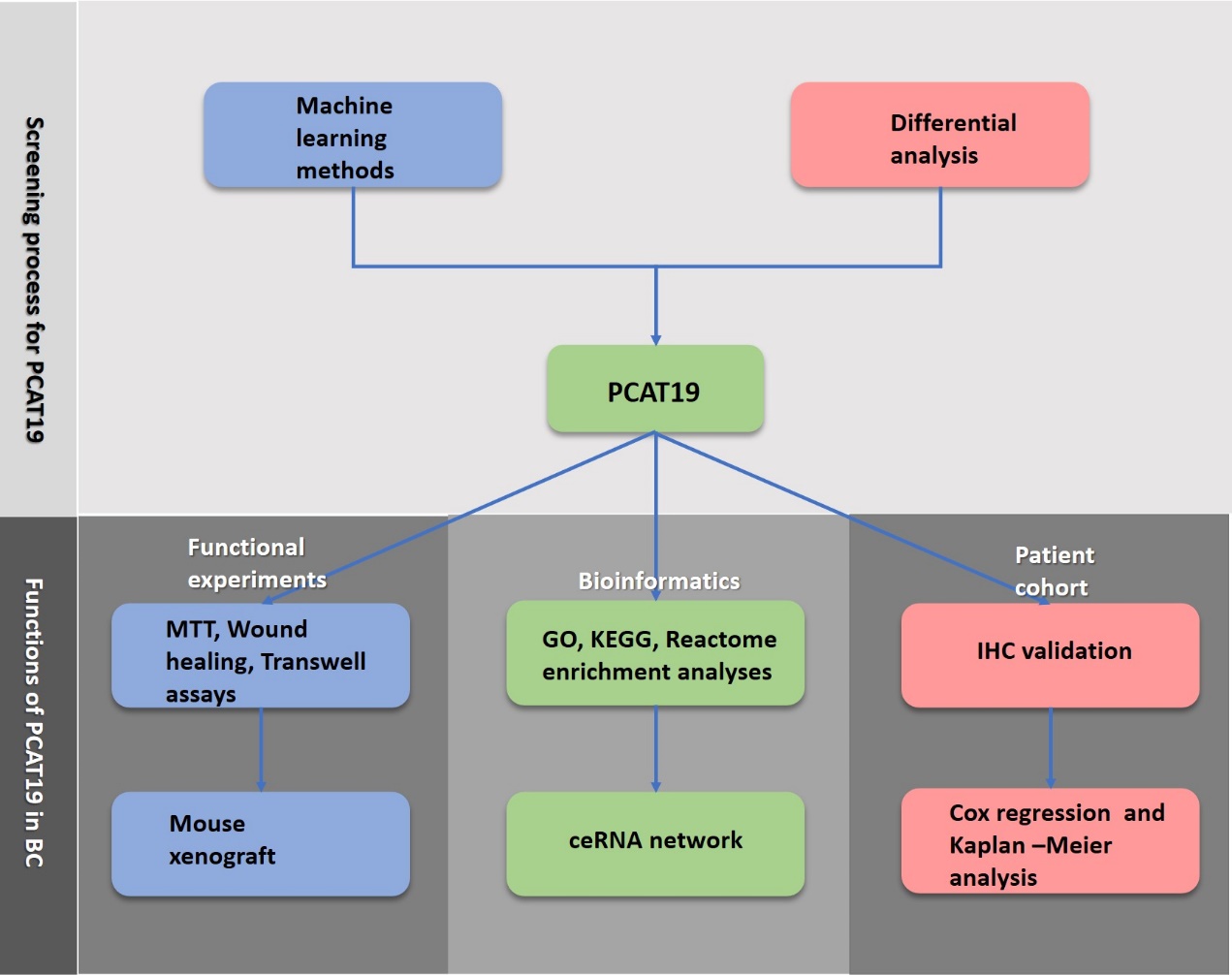


Figure S3 Schematic diagram of the framework for this work
